# Microinjection of Valproic Acid into the Ventrolateral Orbital Cortex Enhances Stress-Related Memory Formation

**DOI:** 10.1371/journal.pone.0052698

**Published:** 2013-01-03

**Authors:** Yan Zhao, Bo Xing, Yong-hui Dang, Chao-ling Qu, Feng Zhu, Chun-xia Yan

**Affiliations:** 1 Department of Forensic Medicine, Key Laboratory of Environment and Genes Related to Diseases of Ministry of Education, Xi'an Jiaotong University School of Medicine, Xi'an, Shaanxi, People's Republic of China; 2 Xi'an Mental Health Center, Xi'an, Shaanxi, People's Republic of China; 3 Department of Physiology and Pathophysiology, Key Laboratory of Environment and Genes Related to Diseases of Ministry of Education, Xi'an Jiaotong University School of Medicine, Xi'an, Shaanxi, People's Republic of China; Florida State University, United States of America

## Abstract

There is collecting evidence suggesting that the process of chromatin remodeling such as changes in histone acetylation contribute to the formation of stress-related memory. Recently, the ventrolateral orbital cortex (VLO), a major subdivision of orbitofrontal cortex (OFC), was shown to be involved in antidepressant-like actions through epigenetic mechanisms. Here, we further investigated the effects of the histone deacetylase inhibitor (HDACi) valproic acid (VPA) on stress-related memory formation and the underlying molecular mechanisms by using the traditional two-day forced swimming test (FST). The results showed that VPA significantly increased the immobility time on day 2 when infused into the VLO before the initial forced swim stress on day 1. The learned immobility response to the stress was associated with increased phosphorylation of extracellular signal-regulated kinase (ERK) in VLO and hippocampus on the first day. The levels of phosphorylated ERK (phospho-ERK) in VLO and hippocampus were significantly decreased when retested 24 h later. The pretreatment with intra-VLO VPA infusion further reduced the activation of ERK on day 2 and day 7 compared with the saline controls. Moreover, the VPA infusion pretreatment also induced a significantly decreased BDNF level in the VLO on day 2, whereas no change was detected in the hippocampus. These findings suggest that VPA enhance the memories of emotionally stressful events and the ERK activity is implicated in stimulating adaptive and mnemonic processes in case the event would recur.

## Introduction

Emotionally stressful events are associated with many psychological disorders, most notably major depression and post-traumatic stress disorder [1], [2]. Memories of stressful experiences are usually strong and longlasting, and thereby can disrupt cognitive and emotional behaviors seriously [3]. The complex interplay between the neocortex and the limbic system (e.g. hippocampus and amygdala) mediates the formation of stress-related memories that are important for adaptation to similar environmental circumstances. The ventrolateral orbital cortex (VLO), a major subdivision of orbitofrontal cortex (OFC), is well known to be involved in nociception [4], [5]. Recently, this brain area has received considerable attentions for its role in antidepressant response in animal models such as forced swimming test (FST) [6], [7].

The FST is an appropriate model to study the underlying neurobiological mechanisms of stress-related memory formation. The immobility response observed 24 h later after the initial test in rat can be viewed as an adaptive learning response to stress [8]. The encoding of these stressful memories requires chromatin remodeling such as histone H3 phosphorylation and acetylation in dentate gyrus granule neurons [9]. Our recent work demonstrated that valproic acid (VPA) reduced the immobility time in the traditional two-day FST when microinjected into the VLO before the retest [10], indicating some effects of VPA on learned responses. Using a similar model, Chen et al. demonstrated that direct infusion of sanguinarine (SA), a selective mitogen-activated protein kinase phosphatase-1 (Mkp-1) inhibitor, into the VLO also decreased the immobility response to forced swim stress [11]. Since Mkp-1 is capable of inactivating extracellular signal-regulated kinase (ERK) [12], it is conceivable that VPA might affect the acquired immobility response through ERK signaling pathway in the VLO.

There is increasing evidence suggesting that chromatin remodeling through histone modifications is a powerful mediator of gene expression that is required for encoding of long-term memory [13]. Histone acetylation contributes to the transcriptional activation process by altering local chromatin structure and facilitating the sequestration of the basal transcriptional machinery. Behavioral studies in rats revealed the psychologically stressful stimulus, such as forced swimming, exposure to a predator or a novel environment, evoke a distinct transcriptional activation including the activation of ERK in dentate gyrus neurons driven by the phosphorylation and acetylation of histone H3 [9]. VPA, a traditional anticonvulsant and mood stabilizer, has effects on stress-related memory in fear conditioning through its function as a histone deacetylase inhibitor (HDACi) [15], whereas brain-derived neurotrophic factor (BDNF) is critical for learning-related synaptic plasticity and for the maintenance of long-term memory. VPA enhances acquisition, extinction and reconsolidation of conditioned fear and affects BDNF gene transcription via epigenetic regulation [16]. In addition, recent studies also demonstrate that HDACi can rescue fear learning deficits in spontaneously hypertensive rats [17] and induce extinction learning in an animal model of impaired extinction [18].

The aim of this present study was to investigate whether microinjection of VPA into the VLO could affect the formation of forced swim stress related memory. We also examined the phosphorylation state of ERK and the expression of BDNF in an attempt to gain further insights into the molecular mechanism underlying memory formation of the stressful events.

## Materials and Methods

### Animal

All experiments were performed with Sprague-Dawley rats (250–320 g), provided by the Experimental Animal Center of Xi'an Jiaotong University. Rats were housed four per cage under standard housing conditions (lights on from 07:30 to 19:30, temperature 22±2°C, relative humidity 60±5%) and had food and water *ad libtum*. The experimental protocols were approved by the Institutional Animal Care Committee of the Xi'an Jiaotong University. All efforts were made to minimize the number of animals used, as well as distress to the animals.

### Bilateral intra-VLO guide cannula placement

At the time of surgery, the weight of the rats was 280±20 g. All rats were anaesthetized with sodium pentobarbital (50 mg/kg, intraperitoneally) and the head was immobilized in a stereotaxic frame. A small craniotomy was performed just above the VLO. A stainless steel guide cannula (0.8 mm in diameter) was stereotaxically inserted (3.2 mm anterior to Bregma, ±2.0 mm lateral, 2.6 mm below cortical surface) and fixed to the skull with dental cement. The dummy cannula was inserted to prevent clogging. Once the animals had recovered from anesthesia, they received sodium penicillin (0.2 million units per day for 4 days, intraperitoneally) to prevent wound and intracerebral infections. After surgery, rats were housed singly and allowed for 7 days to recover before behavioral procedures began.

### Drugs and intra-VLO microinjection

**Figure 1:**
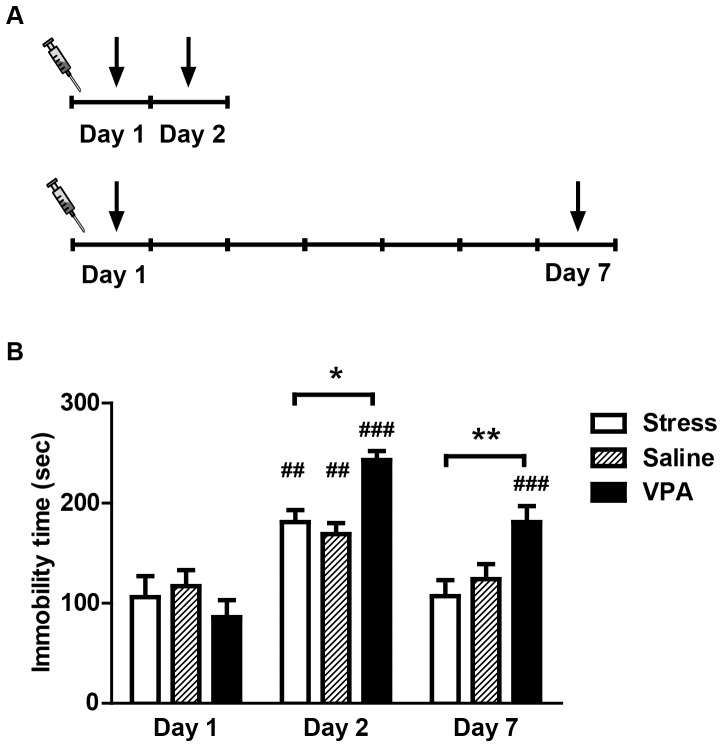
The influence of intro-VLO injection of VPA on forced swim stress induced stress-related memory. A: Time line of the experimental procedure. The syringe indicated that the VPA (300 µg) or saline were bilateral intro-VLO injected 30 min before the initial forced swim stress. The black arrows indicate the exposure of forced swim stress. B: The immobility time at the different test day (day 1, 2 or 7). The immobility time of VPA (300 µg) treated rats were compared with the saline group at the same time point. All data are expressed as the mean ± SEM. **p<0.01 and ***p<0.001 versus pre-test or saline group.

Valproic acid sodium salt were purchased from Sigma-Aldrich (Sigma Chemical, St. Louis, MO, USA) and dissolved in physiological saline at a final concentration of 300 µg/0.5 µl. Rats were randomly divided into 5 groups (Figure 1A): sham group (the rats) (n = 6, underwent sham-operated without microinjection nor forced swim stress); stressed group (n = 24, underwent the forced swim stress without any microinjection treatment); VPA groups (n = 24, microinjection of VPA before initial forced swim stress and test 1 day or 6 day later); valpromide (VPM) (60 µg/0.5 µl/side, the does following the previous study [19] with necessary modification) groups (n = 6, microinjection of VPM before initial forced swim stress and test 1 day later); saline control groups (n = 24, microinjection of saline before initial forced swim stress and test 1 day or 6 days later). During the microinjection, the mice were gently held and the dummy cannula was removed and replaced with injection cannula. Injection cannula was inserted into the brain 2.0 mm deeper that the guide cannula (total depth 4.6 mm). VPA and VPM were slowly infused through a 1.0 µl-microsyringe at a constant speed over 1 minute. Saline of equal volume was injected into the VLO as vehicle control.

### Forced swimming stress

The forced swimming tests were conducted in a quiet, dimly lit room (45 lux) between 19:00 and 21:00. The rats were placed into a plastic container (height 45 cm, diameter 25 cm) with water at a depth of 25 cm (22±2°C), from which they cannot escape. For the first exposure (day 1), 30 min after VPA microinjection, rats were placed into the water for 15 min. For the second exposure (day 2 or day 7), rats were placed into the water for 5 min. Immobility was defined as inability of the animal to perform any movements apart from positioning its head above the surface of water. Behavioral performance was videotaped and analyzed by observers who were unaware of the treatment. To rule out the possible effects of microinjection procedure itself on the behavioral performance, a “stressed” control group (underwent the forced swim stress without any microinjection treatment) was also analyzed.

### Injection site verification

The injection sites were histologically identified or visually confirmed after the behavioral tests or before western blotting (Figure S1). For forced swimming tests on day 1, day 2 or day 7, the drug injection sites were marked by injection of Pontamine Sky Blue dye (0.5 µl, 2% in 0.5 M sodium acetate solution) at the end of the experiment. Under deep anesthesia, the animals were transcardially perfused with 0.9% normal saline, followed by 10% formalin. The brains were then removed and fixed in 10% formalin solution for 7 days. The brains were cut into 30 µm thick sections using a freezing microtome, and then the slices were stained with cresyl violet. The injecting sites were histologically identified within the VLO, with an example shown in Figure S1. For western blotting, rats were sacrificed by rapid decapitation after the last swim test. Brain tissues from VLO and hippocampus were obtained with 1 mm-diameter tissue punch and stored at −80°C until use. The injection sites in VLO were visually identified according to the Paxinos & Watson atlas (1986) (Figure S1). Only rats with the correct bilateral injection sites were included in the behavioral and protein analysis.

### Protein extraction and western blotting analysis

Samples of VLO and hippocampus were homogenized at 4°C with a glass homogenizer, in a RIPA buffer (Cybrdi, Frederick, MD, USA) with a protease and phosphatase inhibitor cocktail (Roche, Basel, Switzerland). The insoluble material was removed by centrifugation at 12,000 rpm for 20 min at 4°C. A BCA protein assay kit (Pierce, Rockland,IL, USA) was used to determine total protein levels for each sample. Equal amounts of protein for each group were separated on a 12% SDS-PAGE gel and transferred to an NC membrane (Millipore Corporation, Bedford, MA, USA). The blots were first probed with antibodies against the phosphorylated forms of the protein and then stripped and probed with antibodies against total proteins of same type. The following antibodies were used, phospho-ERK1/2 (Thr202/Tyr204) (Millipore Corporation, Bedford, MA, USA) at 1∶1000, ERK1/2 (Millipore Corporation, Bedford, MA, USA) at 1∶1000, BDNF (Abcam, Cambridge, UK) at 1∶1000, β-actin (Santa Cruz, California, USA) at 1∶1000, goat anti-rabbit or anti-mouse IgG horseradish peroxidase (HRP)-conjugated secondary antibody (Bioworld, Dublin, OH, USA) at 1∶10000 dilution. β-actin was used as a loading control.

### Data analysis

The data analyses were performed using commercially available software GraphPad Prism 5.0. All data were expressed as the mean ± SEM. The effect of drug treatment and forced swim days on immobility and protein expression in rats were determined using two-way analysis of variance (ANOVA). Where appropriate, Bonferroni's post hoc tests were used to determine differences between groups. For western blot, the difference of protein expression levels between sham and stress-control rats was determined using Student's t test. The expression levels of the sham group were set at 100% and all data were normalized to β-actin. In all cases, *p*-values were two-tailed and comparisons were considered statistically significant at *p*<0.05.

## Results

### Influence of intro-VLO injection of VPA on forced swim stress related memory formation

The immobility time of the rats was affected by both intra-VLO drug treatment (F_2, 45_ = 5.73, *p*<0.01) and by the time factor (F_2, 45_ = 29.97, *p*<0.0001) (Figure 1B). Moreover, a significant interaction was detected between these factors (F_4, 25_ = 4.37, *p*<0.01). Bonferroni post hoc analysis revealed increased immobility time in all three groups on day 2 (stress: *p*<0.01; saline: *p*<0.01; VPA: p<0.0001), when compared with day 1 within the same group. No significant increase in immobility time was observed in stress-control and saline-treated rats on day 7. Injections of VPA before the tests significantly increased the immobility time on day 7 when compared with day 1 within the same group (*p*<0.0001). Furthermore, post hoc test also revealed increased immobility time in the VPA-treated rats than in the stress controls on day 2 (*p*<0.05) and day 7 (*p*<0.01).

Since VPA is also a potent GABAergic signaling enhancer besides a HDACi, we further investigated the effect of valpromide (VPM) that enhance GABAergic signaling but not alter HDAC activity on the immobility time during the two-day FST injected into the VLO before the initial forced swim stress on day 1. The pretreatment with VPM showed no alteration during the whole process compared with saline controls (Figure S2), suggesting the actions of VPA on stress-related memory formation mainly depend on its HDAC inhibitor functions.

### Effect of intra-VLO VPA injection on protein levels of ERK and BDNF in VLO

**Figure 2:**
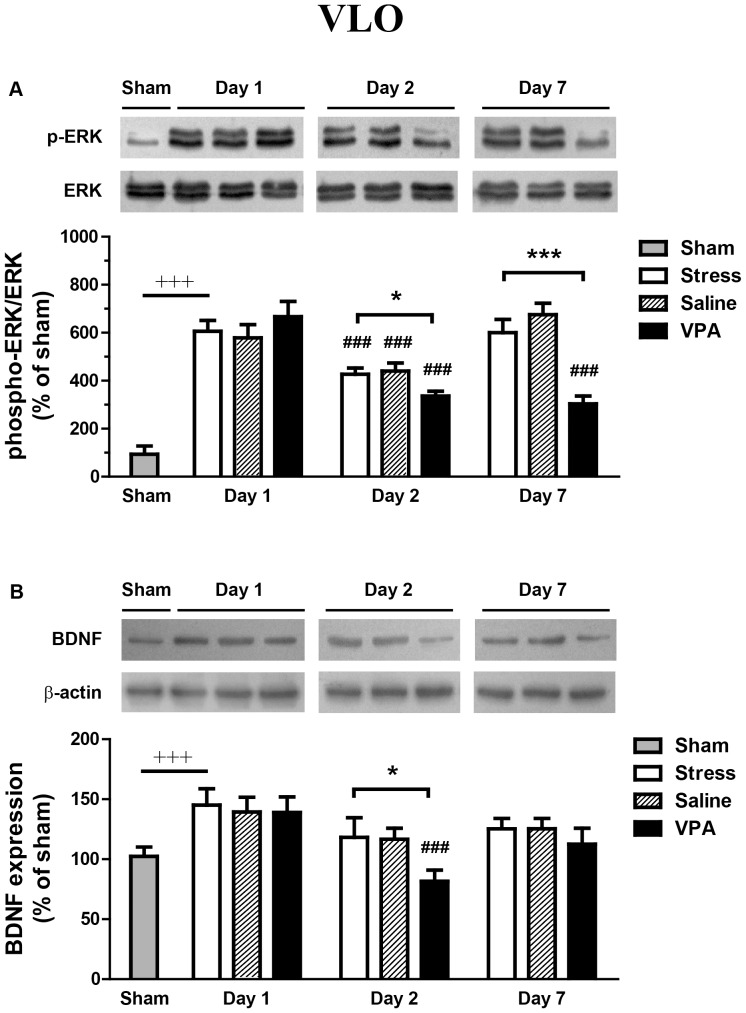
Effect of intra-VLO VPA injection on protein levels of ERK and BDNF in VLO assessed by western blotting. The phosphorylation state of ERK was expressed as phospho-ERK to total-ERK. The BDNF results are normalized to β-actin. Sham group was set as 100%. The data are expressed as the mean ± SEM. ***p<0.0001, difference between sham and saline group on day 1; ^###^ *p*<0.0001, difference from day 1 within the same groups. *p<0.05, ***p<0.0001, difference between VPA and stress groups.

The initital forced swim exposure on day 1 significantly enhanced phospho-ERK expression about 6 fold (stress-control vs. sham, *p*<0.0001) (Figure 2A). A main effect was detected between the treatment (F_2, 45_ = 7.37, *p*<0.01) and time (F_2, 45_ = 18.11, *p*<0.0001) in rats (Figure 2A). A significant interaction was also detected between these factors (F_4, 45_ = 7.49, *p*<0.0001). One day later (day 2), exposure to the forced swim stress again significantly decreased the phosphorylation levels of ERK in VLO in all three groups (stress: *p*<0.0001; saline: *p*<0.0001; VPA: *p*<0.0001, compared with day 1 within the same group). However, when the second forced swim exposure was delayed to six days later (day 7), only previous VPA-treated rats showed obvious decreased in ERK phosphorylation (*p*<0.0001) as compared with VPA-treated rats on day 1. Furthermore, Bonferroni post hoc test also revealed decreased phospho-ERK in the VPA-treated rats than in the stress controls on day 2 (*p*<0.05) and day 7 (*p*<0.0001). Total ERK expression was not changed in any of those groups.

Since it is well known that the phosphorylation of ERK leads to regulation of gene transcription, we examined whether intra-VLO administration of VPA (300 µg/0.5 µl) affects the total protein expression of BDNF. After forced swim stress, Student's t-test revealed significant differences of BDNF expression in VLO region between stress-control group and the non-stressed sham group (*p*<0.0001) (Figure 2B). The level of BDNF expression in the VLO was affected both by VPA treatment (F_2, 45_ = 4.23, *p*<0.05) and by the time after injection (F_2, 45_ = 5.89, *p*<0.01). A significant interaction was also detected between these factors (F_4, 45_ = 7.49, *p*<0.0001). Exposure to the forced swim stress again on day 2 obviously decreased BDNF expression in the VLO in VPA-treated groups (*p*<0.0001, compared with day 1 of VPA group). However, when the second force swim exposure was performed on day 7, rats in all three groups showed no obvious change in BDNF expression as compared with rats on day 1. Furthermore, Bonferroni post hoc test revealed decreased BDNF expression in the VPA-treated rats than in the stress controls on day 2 (p<0.05). But this decrease was not observed on day 7 (*p* = 0.98).

### Effect of intra-VLO VPA injection on protein levels of ERK and BDNF in hippocampus

**Figure 3:**
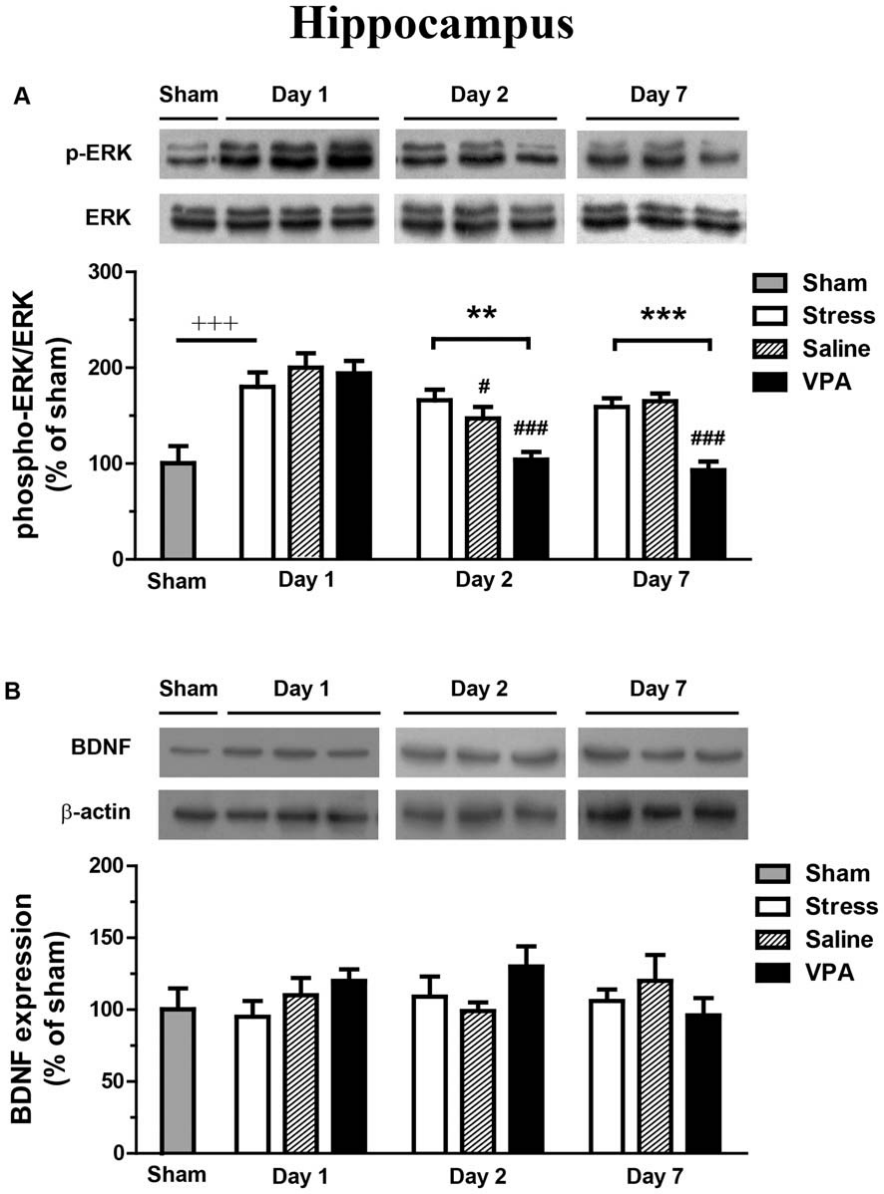
Effect of intra-VLO VPA injection on protein levels of ERK and BDNF in hippocampus. The phosphorylation state of ERK was expressed as phospho-ERK to total-ERK. The BDNF results are normalized to β-actin. Sham group was set as 100%. The data are expressed as the mean ± SEM. ***p<0.0001, difference between sham and saline group on day 1; ^#^ *p*<0.05, ^###^ *p*<0.0001, difference from day 1 within the same groups. **p<0.01, ***p<0.0001, difference between VPA and stress groups.

The expression of the phospho-ERK in the hippocampus was significantly enhanced by the forced swim stress on day 1 (about 1.8 fold) as compared with sham group (*p*<0.0001). Two-way ANOVA detected significant effects of VPA treatment (F_2, 45_ = 11.79, *p*<0.0001) and time (F_2, 45_ = 24.01, *p*<0.0001) as well as an interaction (F_4, 45_ = 4.49, *p*<0.01) on hippocampal phospho-ERK expression in rats (Figure 3A). The phospho-ERK was significantly downregulated in the saline and VPA groups (*p*<0.05 and *p*<0.0001, respectively) on day 2 and in the VPA group on day 7 (*p*<0.0001), as compared with day 1 within the same group. Notably, the post hoc test revealed decreased phosphorylation levels of ERK in VPA-treated rats on day 2 and day 7 (*p*<0.01, *p*<0.0001, respectively), compared with the stress-control groups. Total ERK expression was not changed in any of those groups.

After forced swim stress, Student's t-test revealed no differences of hippocampal BDNF expression between stress-control group and the non-stressed sham group (*p*>0.05) (Figure 3B). The BDNF expression in the hippocampus was not influenced by previous VPA treatment (F_2, 45_ = 0.75, *p* = 0.49) or by the time (F_2, 45_ = 0.16, *p* = 0.55) (Figure 3B).

## Discussion

The present study demonstrated that microinjection of VPA into the VLO enhanced the acquisition of stress-related memory. Animals exposed to immediately forced swim showed an increase in the immobility time on retest, which was strengthened by bilateral microinjection of VPA into the VLO before the initial forced swim session. The enhanced behavioral responses were associated with decreased levels of phospho-ERK and BDNF in the VLO. These findings provide evidence for an involvement of ERK and BDNF signaling in rat VLO during the stress-related memory.

The FST is a widely used behavioral test which predicts the clinical efficacy of antidepressant drugs. When rats or mice are forced to swim in an inescapable cylinder with water, they show a characteristic behavioral response 24 hours later in retest: they decrease their attempts to escape from the water and adopt an immobile posture. According to the initial description by Porsolt et al., the fact that the rat is more immobile on the test day indicates a state of behavioral despair or helplessness in which the animal has learned that escape is impossible and resigns itself to the experimental conditions [16], [20]. Alternatively, among others, the behavioral immobility of rodents is thought to be a consequence of adaptive response to a stressful situation more than “despair” since rats are less fearful on subsequent immersion than on the previous one, the familiarity with the environment rather than “despair” may induce behavioral immobility [21]. Therefore, the characteristic and readily identifiable immobility response could also be regarded as an adaptive learning and memory response with the experimental advantage that it fully develops overnight.

Previous studies have suggested that the memories of emotionally stressful events are dependent on hippocampus and amygdala [22]. The hippocampus supports a specialized system for forming episodic memories of emotional or stressful events, while the amygdala plays an important role in enhancing hippocampus-dependent memory with emotion. Recent evidence has shown that microinjection of the VPA or sanguinarine into the VLO significantly attenuates FST-induced immobility suggesting the role of VLO in mediating antidepressant action [13], [14]. In addition, several lines of evidence have demonstrated that the OFC, including VLO, contributes to reward processing and related memory formation [23], [24]. Electrophysiological data further indicated that the conjunction of the acquired incentive value of stimuli and the use of that information to guide behavior are associated with neuronal activity in the OFC [25], [26]. Behavioral studies in rats are consistent with these findings, as OFC lesions impaired reversal learning in “go, no-go” olfactory discrimination tasks [27], and the bilateral destruction of the VLO produced allocentric (adjacent-arm maze task) but not egocentric (cheeseboard task) spatial deficits [28]. To our knowledge, the results reported here provided first evidence that the VLO is highly involved in the adaptive and mnemonic processes during the stressful event that would re-occur in the future.

VPA is well-established antiepileptic medication that is also commonly used in the acute and chronic management of bipolar disorder. It is found that this anticonvulsant has an effect on mnemonic processes. Memory impairment associated with antiepileptic drug (AED) therapy, including VPA treatment, in children is an important concern given the potential negative effects of treatment on school learning and performance [29]. A recent study has revealed that chronic high dose VPA treatment impairs the ability of rat to perform a hippocampus-dependent spatial memory test (novel object location) and reduces cell proliferation in the hippocampus [30]. However, Pentyl-4n-valproic acid, a derivative of VPA, improves spatial learning and long-term memory for avoidance conditioning [31]. Bredy and colleagues have shown that a single VPA injection can enhance acquisition, extinction, and reconsolidation of conditioned fear in mice [32]. In a previous study, we tested the effects of the microinjection of VPA into VLO before re-test and found indeed that this treatment reduced immobility time in the FST, whereas it did not alter the locomotor activity [10]. In the present study, we have observed an increase of the immobility time in the retest when the VPA was infused into the VLO before pre-test. Thus, given that the microinjection of VPA into VLO has opposite effects on the response to the same forced swim stressor for rats at different time points, it was reasonable to predict that the normal acquisition or retrieval of the stress-related memory would be affected differently by VPA when infused into VLO on pre-test or retest. On one hand, VPA enhances the acquisition of adaptive behavioral immobility in rats, and on the other, it disturbs the retrieval of the learned behavioral response to the forced swim stress.

The precise mechanisms by which VPA brings about these effects in VLO are not well understood. VPA treatment can transiently activate the expression of phospho-ERK expression [33], [34]. However, we did not detect the alteration of phospho-ERK expression on day 1 (formation of the stress-related memory) since the changes may be masked by the stress-induced phospho-ERK elevation [35]. When retest on day 2, the rats which received intra-VLO VPA injection on day 1 exhibited an increased immobility time, indicating enhanced stress-related memory formation caused by the VPA pretreatment on day 1. Moreover, the learned immobility responses on day 2 were associated with decreased phospho-ERK expression (retrieval of the stress-related memory). These results were in line with previous studies that showed that increased immobility time on retest of FST was associated with reduced ERK activation. For example, systemic acute blockade of MEK/ERK signaling produces the increased immobility time in the FST [36]. Decreased ERK phosphorylation is associated with the learned depressive-like actions in hippocampus and PFC following chronic forced swim stress [37]. Furthermore, Chandramohan and colleagues have shown that the epigenetic mechanisms are involved in alteration of ERK activity induced by the forced swim stress in the dentate gyrus because inhibition of ERK activation by using the MAPK-ERK kinase (MEK) inhibitor SL327 blocked the FST-induced increase in histone H3 phospho-acetylation [38]. Most recently, Lin and colleagues have demonstrated that chronic intra-VLO infusion of MS-275, a class I histone deacetylase inhibitor, significantly reduced immobility in the rat FST and tail suspension test (TST) compared with vehicle-treated controls, the effect are associated with an increase in H3 acetylation and elevated CREB and BDNF levels in the VLO [39]. Similar to MS-275, the HDACi VPA, a well-known inhibitor of class I and class II HDACs, enhances the extinction of conditioned fear through its epigenetic regulation of gene expression. Bredy and colleagues have shown that extinction of conditioned fear is accompanied by an increase in histone H4 acetylation around the BDNF P4 gene promoter and increases in BDNF exon I and IV mRNA expression in the prefrontal cortex of mice [19]. Since BDNF is critical for synaptic plasticity, associated with short- or long-term memory formation [40], we measured the BDNF expression of protein by western blotting and found that there was no significant difference between VPA-treated rats and saline-treated controls in the hippocampus when tested on day 2 and on day 7. A decrease in the levels of BDNF was seen in the VLO of VPA-treated rats on day 2, while the difference disappeared when tested on day 7 although the increased immobility in the VPA-treated group was still observed. These results have indicated that the regulation of BDNF signaling could be involved in causing adaptive changes in behavioral plasticity by VPA following forced swim stress but this effect appears to be transient in the VLO. Different stressor can alter BDNF transcription mediated by differential usage of multiple BDNF promoters modified via epigenetic mechanisms to generate region-specific expression and thus behavioral responses to stimuli [41], [42]. It is worthwhile to examine the forced swim stress induced changes in the levels of distinct BDNF transcripts after VPA treatment, although the present study has revealed no marked changes in hippocampal BDNF protein levels.

In summary, the pretreatment with VPA in the VLO renders the learned behavioral immobility response to forced swim stress. The enhanced stress-related memory as measured in the retest might be associated with decreased phosphorylation of ERK in VLO and hippocampus. Further studies examining the epigenetic regulation of BDNF gene will contribute to better understanding of the mechanisms for stress response *in vivo*.

## Supporting Information

Figure S1 Locations of microinjection cannula tips in the VLO of the rats included in the data analyses. Only the data obtained in rats with the tips of injection cannulae located bilaterally in the correct position were included in the statistical analysis. A. For the rats subjected to the behavioral tests, the injection sites were confirmed with Cresyl Violet staining. Photomicrographs of a coronal brain section from a representative rat showed bilateral microinjection sites into the VLO. The section is 30 µm thick. B. For the rats used for Western blotting analysis, the injection sites in VLO were visually identified according to the Paxinos& Watson atlas (1986). Schematic representation shows the approximate location of microinjections into the VLO.
(TIF)

Figure S2 Microinjection of valpromide (VPM) into the VLO has no significant effect on the immobility time compared with the saline group. The rats received saline, VPA (300 µg/0.5 µl/side) or valpromide (60 µg/0.5 µl/side) infusion into VLO on Day 1. On Day 2, all three groups exhibited significantly increased immobility time. The VPA treatment rat group (n = 6) showed a significant increased immobility time compared with saline group (n = 6), whereas no significant difference was found between VPM treatment group (n = 6) and saline controls. ^##^ *p*<0.01, ^###^ *p*<0.001 compared with Day 1 within the same group, *p<0.05 compared with saline group on Day 2.
(TIF)
