# Supplementary figures and images for: Microinjection of Valproic Acid into the Ventrolateral Orbital Cortex Enhances Stress-Related Memory Formation
Source: PLoS One. 2013 Jan 3;8(1):e52698. doi: 10.1371/journal.pone.0052698 (PMC3536774; doi:10.1371/journal.pone.0052698)

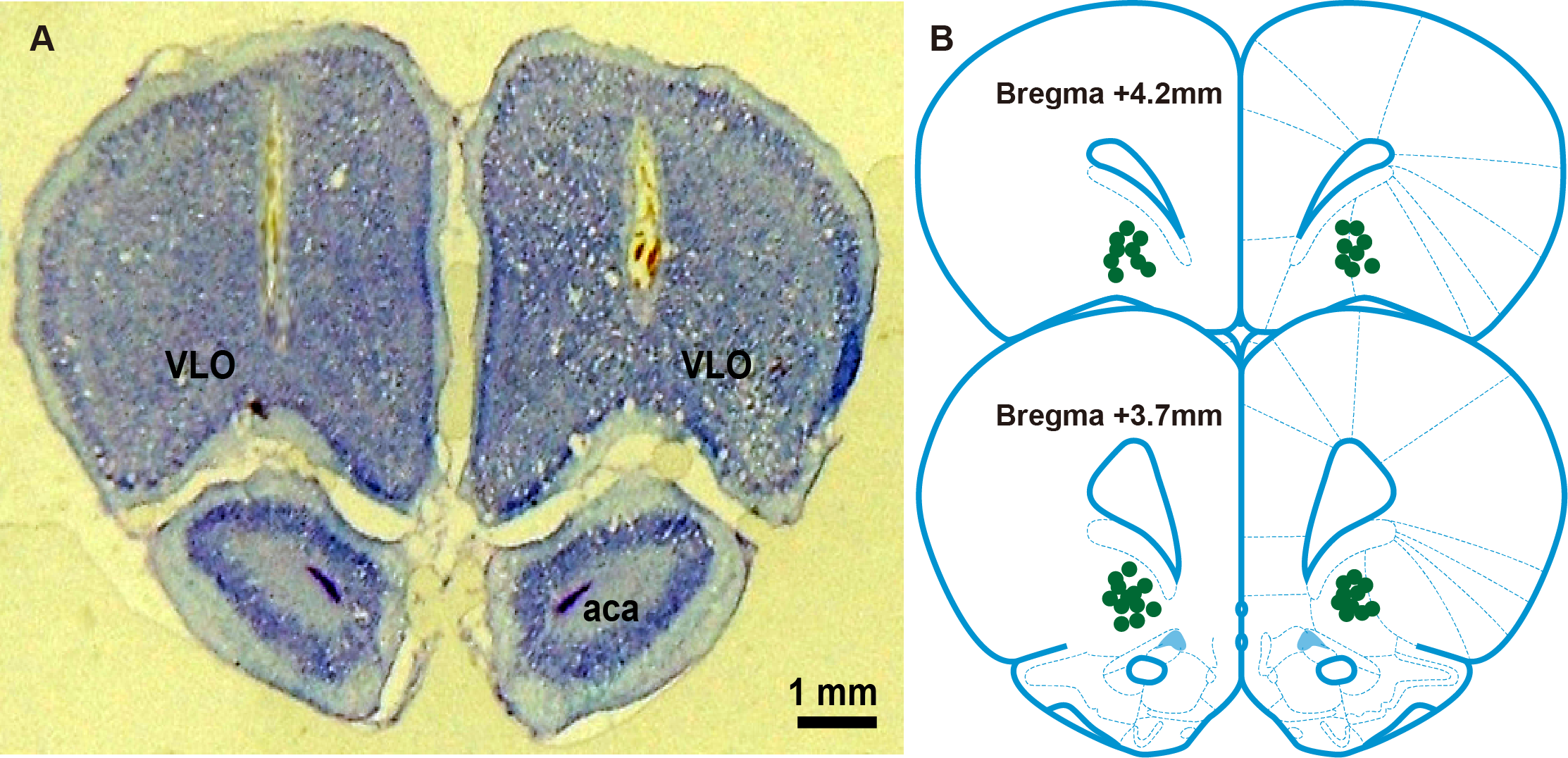

Supplement: Figure S1 — Locations of microinjection cannula tips in the VLO of the rats included in the data analyses. Only the data obtained in rats with the tips of injection cannulae located bilaterally in the correct position were included in the statistical analysis. A. For the rats subjected to the behavioral tests, the injection sites were confirmed with Cresyl Violet staining. Photomicrographs of a coronal brain section from a representative rat showed bilateral microinjection sites into the VLO. The section is 30 µm thick. B. For the rats used for Western blotting analysis, the injection sites in VLO were visually identified according to the Paxinos& Watson atlas (1986). Schematic representation shows the approximate location of microinjections into the VLO. (TIF) [file pone.0052698.s001.tif]

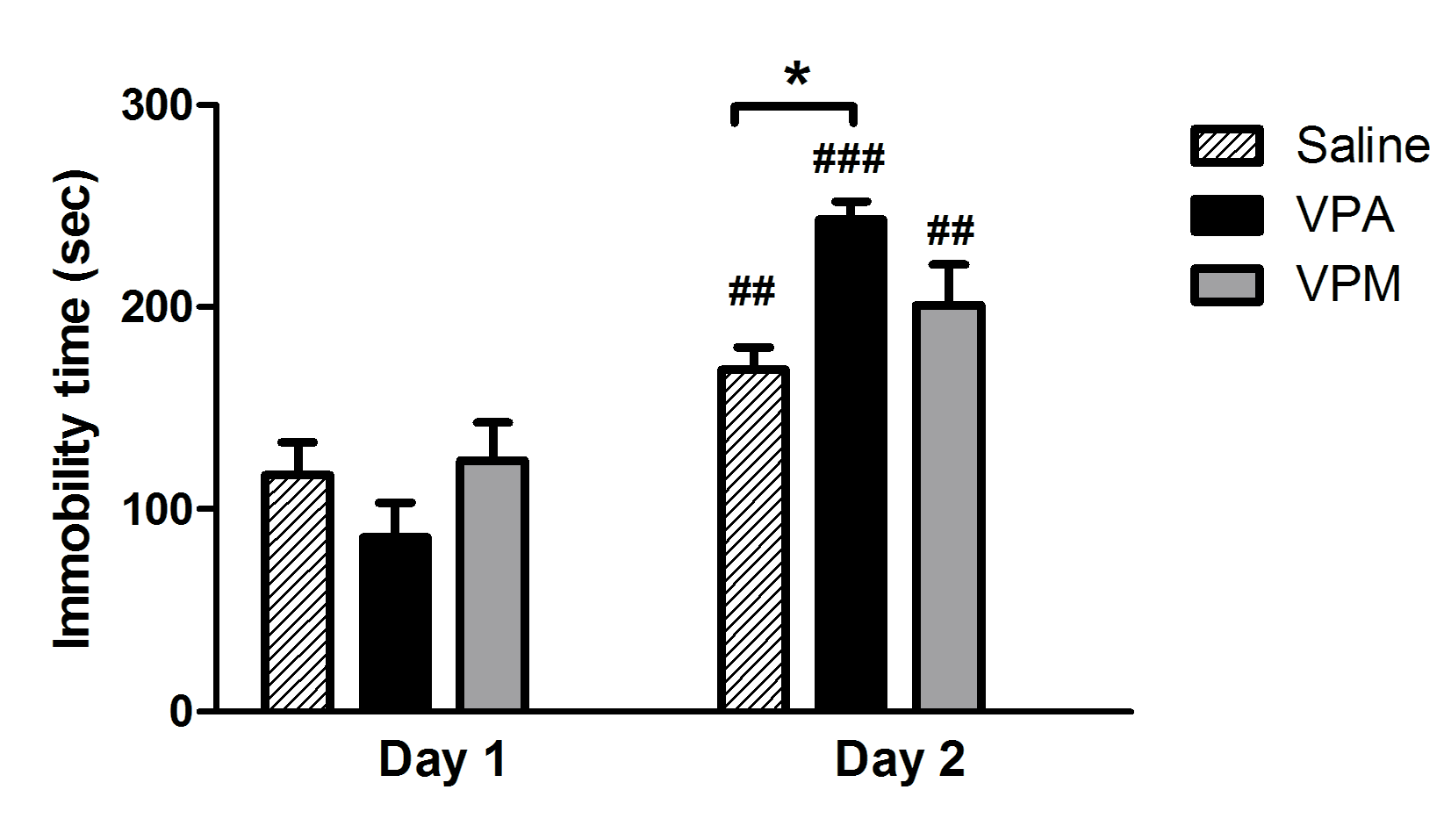

Supplement: Figure S2 — Microinjection of valpromide (VPM) into the VLO has no significant effect on the immobility time compared with the saline group. The rats received saline, VPA (300 µg/0.5 µl/side) or valpromide (60 µg/0.5 µl/side) infusion into VLO on Day 1. On Day 2, all three groups exhibited significantly increased immobility time. The VPA treatment rat group (n = 6) showed a significant increased immobility time compared with saline group (n = 6), whereas no significant difference was found between VPM treatment group (n = 6) and saline controls. ## p<0.01, ### p<0.001 compared with Day 1 within the same group, *p<0.05 compared with saline group on Day 2. (TIF) [file pone.0052698.s002.tif]
